# Pioglitazone Is Associated with Lower Major Adverse Cardiovascular and Cerebrovascular Events than DPP4-Inhibitors in Diabetic Patients with End-Stage Renal Disease: A Taiwan Nationwide Cohort Study, 2006–2016

**DOI:** 10.3390/jcm9113578

**Published:** 2020-11-06

**Authors:** Min-Hao Lin, Huang-Yu Yang, Chieh-Li Yen, Chao-Yi Wu, Chang-Chyi Jenq, George Kuo, Wei-Sheng Peng, Jia-Rou Liu, Ya-Chung Tian, Chih-Wei Yang, Gerard F. Anderson, Lai-Chu See

**Affiliations:** 1 Nephrology Department, Kidney Research Institute, Chang Gung Memorial Hospital at Linkou, College of Medicine, Chang Gung University, Taoyuan 333, Taiwan; n15m25@cgmh.org.tw (M.-H.L.); hyyang01@cgmh.org.tw (H.-Y.Y.); b121919702@cgmh.org.tw (C.-L.Y.); djcc23@cgmh.org.tw (C.-C.J.); mr8393@cgmh.org.tw (G.K.); dryctian@cgmh.org.tw (Y.-C.T.); cwyang@cgmh.org.tw (C.-W.Y.); 2 Department of Health Policy and Management, Johns Hopkins Bloomberg, School of Public Health, Baltimore, MD 21205, USA; ganderson@jhu.edu; 3 Division of Allergy, Asthma, and Rheumatology, Department of Pediatrics, Chang Gung Memorial Hospital at Linkou, Chang Gung University College of Medicine, Taoyuan 333, Taiwan; joywucgu@cgmh.org.tw; 4 Department of Public Health, College of Medicine, Chang Gung University, Taoyuan 333, Taiwan; weisheng@cgmh.org.tw (W.-S.P.); jiarou@mail.cgu.edu.tw (J.-R.L.); 5 Division of Rheumatology, Allergy and Immunology, Department of Internal Medicine, Chang Gung Memorial Hospital at Linkou, Taoyuan 333, Taiwan; 6 Biostatistics Core Laboratory, Molecular Medicine Research Center, Chang Gung University, Taoyuan 333, Taiwan

**Keywords:** Pioglitazone, DPP4-inhibitor, ESRD, cardiovascular outcome, mortality

## Abstract

While pioglitazone reduces insulin resistance and hepatic gluconeogenesis effectively in patients with type 2 diabetes mellitus (T2DM), these benefits remained controversial in patients with end stage renal disease (ESRD). We compared major adverse cardiac cerebrovascular events (MACCEs) and mortality (overall, infection-related, and MACCE-related) of pioglitazone to that of dipeptidyl peptidase 4 inhibitors (DPP4-inhibitors) in patients with T2DM and ESRD. From Taiwan’s national health insurance research database (NHIRD), 647 pioglitazone users and 6080 DPP4-inhibitors users between 1 April 2006 and 31 December 2016 were followed from the 91th date after the ESRD certification until the study outcomes, independently; withdraw from the NHI program, death, or 31 December 2017, whichever came first. After weighting, risks of MACCEs (10.48% vs. 12.62% per person-years, hazard ratio (HR): 0.85, 95% (CI): 0.729–0.985) and all-cause mortality (12.86% vs. 13.22% per person-years, (HR): 0.88, 95% (CI): 0.771–0.995) are significantly lower in pioglitazone group. Subgroup analysis found lower MACCEs risk in the pioglitazone users without insulin therapy (6.44% vs. 10.04% (HR): 0.59, 95% (CI): 0.42–0.82) and lower MACCEs related death (2.76% vs. 3.84% (HR): 0.61, 95% (CI): 0.40–0.95) in the pioglitazone group with dyslipidemia, when comparing with DPP4-inhibitors users. Pioglitazone is associated with lower all-cause mortality and MACCEs in diabetic patients with ESRD, compared to DPP4-inhibitors. These benefits were even more significant in the non-insulin users and patients with dyslipidemia.

## 1. Introduction

Patients with end stage renal disease (ESRD) had poor prognosis, which was caused by the high mortality rate associated to atherosclerosis and infection [1,2,3,4]. Type 2 diabetes mellitus (T2DM) accounts for the great majority of cause to end stage renal disease (ESRD) throughout the world, especially in Taiwan [5,6]. Moreover, the co-existing of T2DM among patients undergoing maintenance dialysis strongly increased the risk of cardiovascular events, including myocardial infarction and cerebrovascular events in comparison to ESRD patients without DM [1,2,3]. In other words, good control of T2DM may prevent patients with ESRD from these fatal events [2,3].

In the population of ESRD, several glucose-lowering agents were of concern. For instance, metformin is contraindicated in patients with advanced chronic kidney disease due to the risk of lactic acidosis [7,8]. Glipizide is the only sulfonylurea that could be prescribed in patients with ESRD, yet it may increase the risk of hypoglycemia and cardiovascular mortality [8,9]. Sodium-glucose transport protein 2 inhibitors (SGLT2 inhibitors) act as a glucose lowering agent via inhibition of glucose reabsorption and it is not suggested for patients with eGFR less than 45 mg/dL [8,9].

In contrast, dipeptidyl peptidase 4 inhibitors (DPP-4 inhibitors) have demonstrated safety and capability as a hypoglycemic agent for patients with ESRD [7,10,11,12]. DPP4-inhibitors can treat hyperglycemia by protecting activation of incretin to induce glucose metabolism, without the side effect of hypoglycemia [13]. DPP4-inhibitors have become the oral hypoglycemic agent (OHA) with the least adverse effect and were frequently prescribed in T2DM patients with ESRD [14,15].

On the other hand, the benefit of pioglitazone remained controversial when it comes to patients with ESRD. Pioglitazone is a thiazolidinedione (TZD), targeting the peroxisome proliferator-activated receptor gamma (PPAR-γ), that can reduce insulin resistance and hepatic gluconeogenesis [16,17,18,19,20]. This agent kept its ability in the setting of renal failure [16,17,21]. Moreover, numerous studies among PPAR-γ showed its potential role in the treatment of cardiovascular disease [16,22,23,24]. These were compatible with the outcome trial and observation study of pioglitazone [21,25,26,27,28,29,30,31]. However, it is of some concern in the population with advanced renal impairment, due to the side effect of fluid overload and the risk of bone loss and congestive heart failure [9,18,21,31,32].

To the best of our knowledge, there was no direct evidence for the benefit of pioglitazone to reduce adverse effects and mortality rate in the category of T2DM with ESRD in comparison to DPP4-inhibitors. Hence, we aimed to estimate the rates of major adverse cardiac and cerebrovascular events (MACCEs) and mortality (overall, infection-related, and MACCE-related) in the diabetic patients with ESRD receiving pioglitazone in Taiwan. The control group was diabetic patients with ESRD receiving DPP4-inhibitors rather than pioglitazone.

## 2. Materials and Methods

### 2.1. Data Source

The primary data source was from the Taiwan national health insurance research database (NHIRD) and Taiwan Death Registry (TDR). The Taiwan National Health Insurance program was founded in 1995 and covered more than 99.6% of individuals since 1997 [33]. Registration data (year of birth, sex, income, place of residence, occupation, date in and out of the NHI program), and original claims for reimbursement (dates of clinical visits, medical diagnoses, medical expenditure, details of prescriptions, examinations, and procedures) are stored in NHIRD. The disease diagnoses were coded using the ICD-9-CM and were switched to ICD-10-CM after 2016. The TDR had the information about the date of death, cause of death (underlying and immediate) for deceased Taiwanese residents. The cause of death was also coded using the ICD-9-CM and were switched to ICD-10-CM after 2008.

Note that both the NHIRD and the TDR are available for research purpose after the identification information were encrypted. The link between these two datasets is feasible because of using the same encryption algorithm. To further protect the privacy of the beneficiaries, the use of NHIRD is restricted at Health and Welfare Data Science Center, Ministry of Health and Welfare (HWDC-MHW), Taiwan and its sub-centers, and only summary results are allowed to carry out from the center.

This study had obtained an approval from the Institutional Review Board of Chang Gung Medical Foundation (approval number: 201900840B0) and the National Health Insurance Administration, Department of Health and Welfare, the holder of the NHIRD.

### 2.2. Study Design

Using the NHIRD and TDR, we designed a nationwide retrospective cohort study with patients having T2DM and ESRD and divided in two study groups: pioglitazone and DPP4-inhibitors. The active control group of DPP4-inhibitors, including sitagliptin, saxagliptin, and linagliptin, allow us to reduce the channeling bias (also called cofounding by indication) [34]. The cohort was followed from the index date till primary outcomes, secondary outcomes, independently, withdraw from the NHI program, death, or 31 December 2017, whichever came first.

### 2.3. Patient Selection

**Figure 1:**
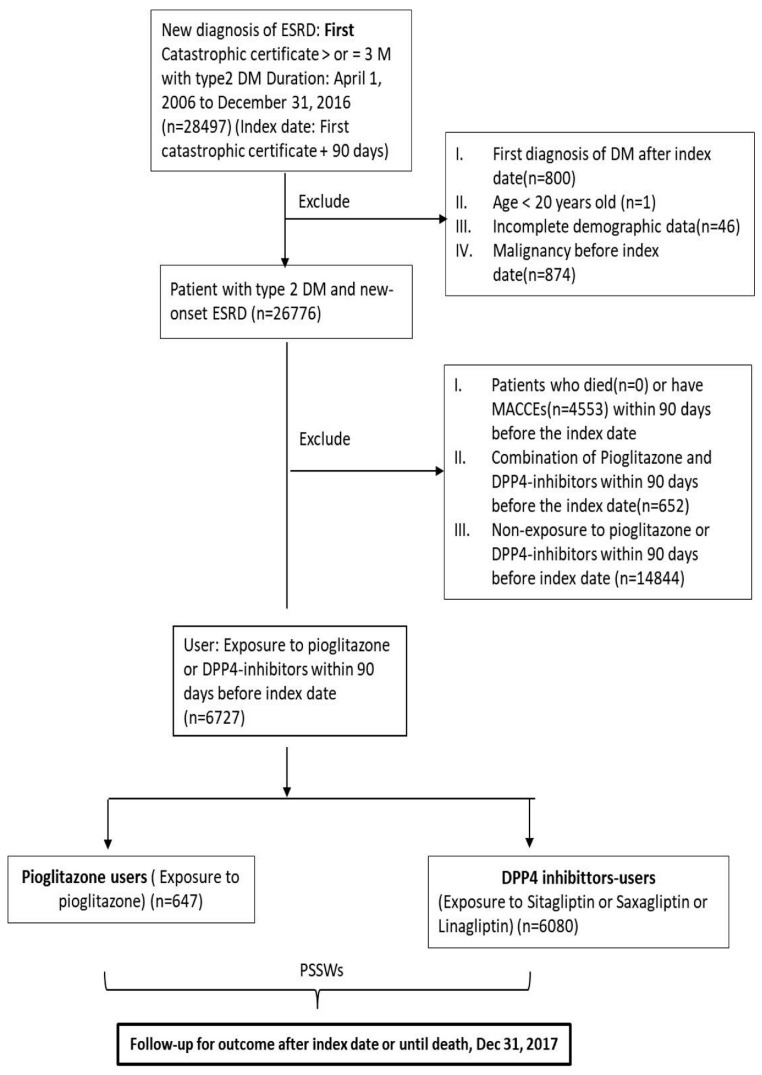
Flow chart of study patient enrollment. DM, diabetes mellitus; DPP4-inhibitor, Dipeptidyl peptidase 4 inhibitor; ESRD, end stage renal disease; MACCEs, major adverse cardiac cerebrovascular events.

The algorithm of patient selection in this study is shown in Figure 1. Patients older than 20 years old with first catastrophic certification of ESRD between 1 April 2006 and 31 December in 2016 and having T2DM were identified as new onset ESRD cohort. The 91th date after the certification was defined as the index date. Patients with newly diagnosed T2DM after index date, patients with malignancy before index date and patients with incomplete demographic data were excluded. Patients who died or had MACCEs within 90 days before the index date were excluded because the events were less likely due to the exposure of pioglitazone or DPP4-inhibitors.

### 2.4. Exposure

All participants in this cohort study were exposed to at least one OHA between ESRD date and index date, either pioglitazone or DPP4-inhibitors. Patients who did not receive either one of these two drugs or who took both drugs were not enrolled.

### 2.5. Covariates and Outcomes

We considered the following covariates: (1) demographic characteristics (age, gender, income level, and place of residence), (2) comorbidities within one year before index date (hypertension, dyslipidemia, liver cirrhosis, connective tissue disease, atrial fibrillation, and peripheral arterial disease), (3) hospitalization history (heart failure, myocardial infarction, stroke, and infection) within 3 years before index date, and (4) medication within 90 days before index date (ACEi or ARB, other anti-HTN, diuretics, aspirin or plavix, NSAIDs, insulin, sulfonylurea, acarbose, meglitinides, GLP-1, and anti-cholesterol). To reduce misclassification, all comorbidities had to be at least two visits at outpatient or one hospitalization. Charlson’s score, which weighted based on 14 diseases, was also presented [35].

All-cause mortality as well as MACCEs, including myocardial infarction, cardiogenic shock, new-onset heart failure, coronary revascularization, fulminant arrhythmia, and cerebrovascular events) were the two primary outcomes of this study. The secondary outcomes were infection-related death and MACCEs related death, which were the two most leading cause of mortality in this population. Death due to MACCEs or infection were recognized by surveillance of final diagnosis appertained to hospitalization or emergency room visits, or the underlying cause of death in TDR.

Please see the Supplementary Table S1 for the ICD-9-CM and ICD-10-CM for the study outcomes and covariates for this study.

### 2.6. Statistical Analysis

We used the propensity score method with stabilized weights (PSSWs) to balance the covariates at index date between the two drug groups [36]. The PSSWs provide an appropriate estimate of the main effect variance without compressing or magnifying the sample size of the original data, hence, the designated type I error was maintained. We included the covariates (except Charlson’s score) at baseline (Table 1) in the generalized boosted model (GBM) to obtain PSSWs, because Charlson’s score included some comorbidities and hospitalization history used in this study. The GBM is less affected by large weights and can achieve the optimal balance between the two drug groups, by automatically including interactions or polynomial terms of the covariates [37]. We used the absolute standardized mean difference (ASMD) to examine the balance of covariates at index date between the two drug groups, because balance is a property of the sample and not of an underlying population. The value of ASMD ≤ 0.1 indicated a negligible difference in covariates between the two study groups [38]. We computed the incidence rates as the total number of study outcomes during the follow-up period divided by person-years at risk. We assessed the hazard ratio (HR) of study outcomes for pioglitazone versus DPP4-inhibitors using survival analysis (Kaplan–Meier method and log-rank test for univariate analysis and Cox proportional hazards model for multivariate analysis). We also performed subgroup analysis and used forest plot to show whether the pioglitazone group had a consistent HR for pioglitazone when compared with the DPP4-inhibitors group in specific subgroups. To maintain a balance of varied covariates between the two drug groups, we re-conducted PSSWs for each subgroup analysis. The significant level of this study was 0.05. All statistical analyses were performed using SAS ver. 9.4 (SAS Institute, Cary, NC, USA).

## 3. Results

### 3.1. Patient Characteristics

There were 28,497 patients with type 2 DM and newly diagnosed ESRD during 1 April 2006 to 31 December 2016 in Taiwan. After excluding those had first diagnosis of T2DM before index date (*n* = 800), age under 20 years old (*n* = 1), incomplete demographic data (*n* = 46), malignancy before index date (*n* = 874), patients who died (*n* = 0) or have MACCEs (*n* = 4553) within 90 days before the index date, took both pioglitazone and DPP4-inhibitors within 90 days before the index date (*n* = 652), did not take either pioglitazone or DPP4-inhibitors within 90 days before index date (*n* = 14,844), there were 647 patients in the pioglitazone group and 6080 patients in the DPP4-inhibitor group (Figure 1). Table 1 illustrated the demographic characteristics, comorbidities, hospitalization history, and use of medication between the two drug groups. Before PSSWs, there were more female, rural resident, hospitalization history of heart failure, use of angiotensin-converting enzyme inhibitors (ACEi) or angiotensin II receptor blockers (ARBs), and use of other oral hypoglycemic agent (OHA) in the pioglitazone group than the DPP4-inhibitor group. After PSSWs, all covariates were balanced between the two drug groups as ASMDs were less than 0.1, except the use of insulin. This may indicate pioglitazone was prescribed in combination with insulin more frequently and less OHA in comparison with DPP4-inhibitors in our study cohort.

### 3.2. Outcomes

**Figure 2:**
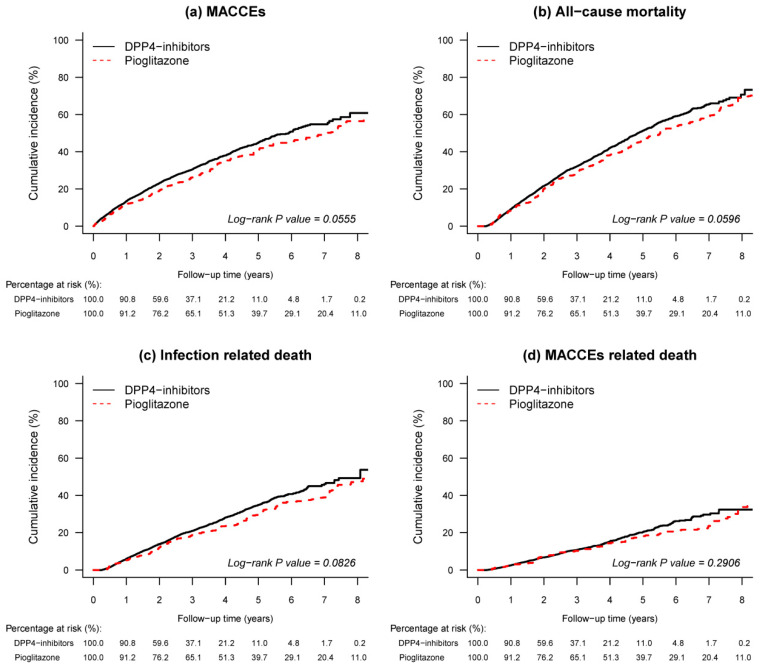
Kaplan–Meier curve of cumulative incidence for primary and secondary outcomes after propensity score stabilize weighting (PSSW). (**a**) Major adverse cardiac cerebrovascular events; (**b**) All-cause mortality; (**c**) Infection related death; (**d**) Major adverse cardiac cerebrovascular events (MACCEs) related death. DPP4-inhibitors, dipeptidyl-peptidase 4 inhibitors.

The incidence rate of study outcomes between the two drug groups are shown in Table 2 and the cumulative incidence vs. follow-up time are plotted in Figure 2 (after PSSWs) and Supplemental Figure S1 (before PSSWs), respectively. Before PSSWs, pioglitazone group had the lower risk of both all-cause mortality (13.07% vs. 13.23% per person-years, (HR): 0.88, 95% (CI): 0.78–0.98) and major adverse cardiovascular events (10.18% vs. 12.64% per person-years, (HR): 0.82, 95% (CI): 0.71–0.95) than the DPPi group. After PSSWs, the risk of MACCEs (10.48% vs. 12.62% per person-years, (HR): 0.85, 95% (CI): 0.73–0.99) and all-cause mortality (12.86% vs. 13.22% per person-years, (HR): 0.88, 95% (CI): 0.771–0.995) remained lower in the pioglitazone group than the DPP4-inhibitor group. Death related to infection or MACCEs were further investigated, which implied an insignificant lower risk in pioglitazone group than the DPP4-inhibitor group after PSSWs (7.42% vs. 7.98% per person-years, (HR): 0.85, 95% (CI): 0.721–1.006 for infection related death; 4.20% vs. 4.04% per person-years, (HR): 0.88, 95% (CI): 0.70–1.10 for MACCEs related death).

### 3.3. Subgroup Analysis

**Figure 3:**
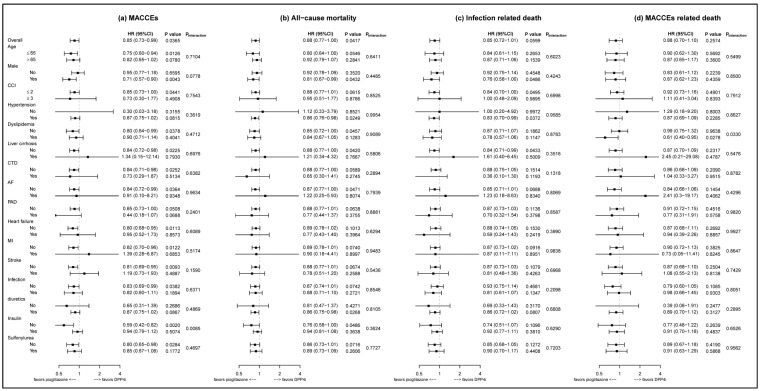
Forest plot of subgroup analysis for primary and secondary outcomes. (**a**) Major adverse cardiac cerebrovascular events; (**b**) All-cause mortality; (**c**) Infection related death; (**d**) Major adverse cardiac cerebrovascular events (MACCEs) related death. AF, atrial fibrillation; CCI, Charlson’s cormobidity index; CI, confidence interval; CTD, connective tissue disease; DPP4i, dipeptidyl-peptidase 4 inhibitors; HR, hazard ratio; MI, myocardial infarction; PAD, peripheral artery disease.

Figure 3 presents the result of subgroup analysis. For MACCEs, the lower HRs were consistently seen in the pioglitazone than in the DPP4-inhibitors group in most subgroups. Noted that patient without insulin therapy had lower risk of MACCEs in the group of pioglitazone than in the DPP4-inhibitors group (6.44% vs. 10.04% per person-years; (HR): 0.59, 95% (CI): 0.42–0.82). This effect was not seen in subjects receiving insulin therapy (interaction *p* = 0.0085). For all-cause mortality and infection related death, the pioglitazone group had a consistent lower HR when compared with the DPP4-inhibitors group in specific subgroups. For MACCEs-related death, pioglitazone group had lower risk (2.76% vs. 3.84% per person-years; (HR): 0.61, 95% (CI): 0.40–0.95) than the DPP4-ihibitor group among patients with underlying dyslipidemia, but such reduced risk was not observed in patients without dyslipidemia (interaction *p* = 0.0330).

## 4. Discussion

In this nationwide cohort study investigating patients with coexisting T2DM and ESRD, the pioglitazone group was associated with reduced MACCEs and all-cause mortality when compared to the DPP4-inhibitor group after PSSWs. These significant findings were not shown clearly in previous real-world study. As one may criticize that saxagliptin had been reported to increase heart failure related hospitalization [39], we also conducted an additional analysis for MACCEs and MACCE-related death which excluded saxagliptin users in control group. The result (Supplementary Table S2) revealed that excluding saxagliptin users from control group did not revoke the effect of pioglitazone to reduce MACCEs. There was no difference of MACCEs-related death between these two groups. Another difference in the subgroup analysis of insulin therapy was that DM patients who were insulin-free were more likely to benefit from pioglitazone with lower MACCEs. Furthermore, patients with underlying dyslipidemia were associated with lower MACCEs related death in pioglitazone users’ group.

Pioglitazone is a full PPAR-γ agonist, affecting multi-system with the potential to promote health or reduce lethal consequences in patients with DM [16,17,18,25,27,29,40]. The advantages and mechanism of PPAR-γ agonist includes inhibition of cytokine production by macrophages, reduction of oxidative stress, improving insulin resistance, control dyslipidemia due to the regulation of adipogenesis, and lowering blood pressure via vasodilation [22,24]. The aforementioned properties may then result in protecting effects, mostly against ischemic stroke and cardiovascular events. Considering the high risk of cerebrovascular accident and cardiovascular events and related mortality, it is not surprising that pioglitazone could eventually decrease the risk of mortality rate among patients with diabetes and ESRD [16,17].

It was not clearly unstressed why patients without insulin therapy had lower risk of MACCEs in the group of pioglitazone than in the DPP4-inhibitors group. However, it was consistent with previous large retrospective study in ArMORR cohort [41]. In our opinion, insulin may increase the risk of hypoglycemia in dialysis patients that affected the compliance of combined OHA [42]. Moreover, considering the phenomenon of “Burnt-Out Diabetes” [43] after progression into ESRD, the use of insulin in dialysis cohort may represent a relative higher HbA1c or more fluctuated serum glycemic status. In addition, insulin per se could be responsible for the increased MACCEs risk in dialysis patient with or without the combination of other glycemic control agent [44]. All these factors could interfere with the effect of pioglitazone for reduced MACCEs. On the other hand, pioglitazone’s effect in lipid lowering via regulation of adipogenesis remained intact in ESRD patients [16,29], which could be supported by our observation that patients with underlying dyslipidemia benefit more from pioglitazone rather than DPP4-inhibitors.

There were plenty of clinical studies which were designed to investigate the effectiveness and safety of pioglitazone, such as PROactive trial [25,40], CHICAGO trial [45], and PERISCOPE trial [46], whether compared to placebo or other oral glucose lowering agents. The PROactive trial investigated DM patients with prior macrovascular events and revealed a reduction of all-cause mortality, non-fatal myocardial infarction, and stroke in the intervention group [25,40]. The CHICAGO trial displayed the role of pioglitazone to slow progression of carotid artery intima-media thickness [45] while the PERISCOPE trial proved the effect of pioglitazone to lower coronary atherosclerosis in DM patients with a history of coronary artery disease [46]. None of these clinical trials discussed the effect and safety outcome in the setting of end stage renal disease. In contrast, some clinicians compared pioglitazone to placebo or other OHA in the population with ESRD [21]. However, these trials were either too short (most had mean follow up less than 1 year) or too small (participants were less than 100 in most studies) to provide solid evidence. One large retrospective study conducted by Brunelli et al. used the data extracted from the ArMORR cohort had been published in 2009 [41]. This study, comparing pioglitazone with placebo among patients receiving incident hemodialysis, consistently disclosed the effect of reduction in all-cause mortality in the group of non-insulin participants. This effect was contributed to non-CV mechanisms, explained by Brunelli and associates. Unlike the former research, the current study pointed out a significant reduction of both all-cause mortality and MACCEs. However, with similar design, our study offered more robust evidence through the setup of control group and a long follow-up duration.

One of the obstacles to obtain diabetes control in the ESRD population is that there are few OHA that are safe and effective in these patients. As mentioned above, DPP4-inhibitors maintained their effectiveness in diabetic patients with renal impairment and even ESRD [7,10,47]. Some studies indicated that specific DPP4-inhibitors may lead to adverse cardiovascular events, such as recurrent myocardial infarction and hospitalization, due to heart failure in selected populations [39,48], while the others take a positive attitude among cardiovascular outcome in patients with ESRD [14,49]. Overall, DPP4-inhibitors are thought to be neutral with regard to the cardiovascular events in DM population [12,50]. Based on this feature, patients who received DPP4-inhibitors were enrolled as the control group in our study. The results of present study demonstrated a reduction in all-cause mortality and MACCEs in the pioglitazone group. This finding implied the safety of pioglitazone in patients with ESRD and may further point out the possible benefit of pioglitazone in the treatment of DM-ESRD patients beyond glycemic control.

Unfortunately, pioglitazone had been reported to be associated with several adverse events. Among these side effects, edema, weight gain, and heart failure are of concerned to many clinicians, although there is some controversy [9,31,32]. In fact, these side effects were mostly reported by research in populations with normal renal function to mild renal impairment. For patients under maintenance dialysis, it is likely that the edema and weight gain can be controlled by adjustment of the dialysis modality. However, the possibility to increase burden of ultrafiltration, particularly in peritoneal dialysis population, may require further investigation. The true incidence and influence of these adverse events, especially edema, weight gain, and heart failure, in the ESRD cohort with pioglitazone therapy remained unclear and thus further randomized control trials are required in future.

There were several limitations in our study. First of all, this cohort study was not assembled to compare the efficacy of the glucose lowering of these two different OHA. In other words, the present study did not examine the glucose control ability of these two agents. Second, the NHIRD contains most information which was required for the purpose of reimbursement. However, the absence of laboratory results (i.e., glycohemoglobin), examination findings (i.e., left ventricular ejection fraction), and lifestyle characteristics (i.e., body mass index and cigarette smoking) do not allow us to examine these particular risk factors. Third, this research is conducted retrospectively in an observational perspective, which means the indication, the dosage, and the compliance in each group were not standardized. To mitigate this shortcoming, PSSW was applied with as much covariates as available in this administrative database, including socioeconomic status, comorbidities, and medication use. Fourth, the side effects, as discussed previously, and minor events including hypoglycemia were hard to obtain in both groups. Therefore, the findings of this study should be interpreted with caution and a further prospective randomized control trial is warranted.

In summary, the current study provided robust evidence to support that pioglitazone is associated with lower all-cause mortality and MACCEs in comparison to DPP4-inhibitors in diabetic patients with ESRD, especially in those non-insulin enrollees. Besides, patients with dyslipidemia are more likely to benefit from pioglitazone among MACCEs related death.

## Figures and Tables

**Table 1:** Baseline characteristics of diabetic patients with end-stage renal disease.

	Before PSSW					After PSSW				
	Pioglitazone (*n* = 647)		DPP4i (*n* = 6080)		ASMD	Pioglitazone (*n* = 647)		DPP4i (*n* = 6080)		ASMD
	*n*	(%)	*n*	(%)		*n*	(%)	*n*	(%)	
Age (year)										
Mean ± SD	64.64	±11.75	65.07	±12.50	0.0352	64.98	±11.00	65.05	±12.43	0.0054
<65	308	(47.60)	2916	(47.96)	0.1119	264.1	(48.19)	2906.5	(47.96)	0.0278
≥65	339	(52.39)	3164	(52.04)		283.9	(51.81)	3153.7	(52.04)	
Gender										
Male	299	(46.21)	3171	(52.15)	0.1191	282.3	(51.51)	3130.7	(51.66)	0.0029
Female	348	(53.79)	2909	(47.85)		265.7	(48.49)	2929.5	(48.34)	
Income level										
≥25,000	58	(8.96)	558	(9.18)	0.0776	45.4	(8.28)	553.9	(9.14)	0.0886
15,000–25,000	167	(25.81)	1464	(24.08)		137.9	(25.16)	1466.8	(24.20)	
<15,000 or dependent	422	(65.22)	4058	(66.75)		364.8	(66.56)	4039.5	(66.66)	
Place of residence										
Urban	141	(21.79)	1626	(26.74)	0.1497	149.4	(27.27)	1596.7	(26.35)	0.0498
Suburban	163	(25.19)	1590	(26.15)		142.0	(25.91)	1579.8	(26.07)	
Rural	343	(53.02)	2864	(47.10)		205.4	(37.48)	2363.2	(39.00)	
Missing	74	(11.44)	506	(8.32)		51.2	(9.34)	520.2	(8.59)	
Charlson comorbidity index										
0	357	(55.18)	3015	(49.59)	0.0945	305.5	(55.74)	3019.2	(49.82)	0.1106
1	191	(29.52)	2018	(33.19)		163. 1	(29.76)	2006.6	(33.11)	
2	69	(10.66)	741	(12.19)		54.2	(9.90)	734.0	(12.11)	
3	25	(3.86)	243	(4.00)		22.0	(4.01)	238.3	(3.93)	
4+	5	(0.78)	63	(1.03)		3.3	(0.59)	62.2	(1.03)	
**Comorbidities (within 1 year before index date)**										
Hypertension	617	(95.40)	5835	(96.00)	0.0298	525.9	(96.00)	5815.5	(96.00)	0.0000
Dyslipidemia	284	(43.90)	2691	(44.30)	0.0073	245.7	(44.80)	2678.7	(44.20)	0.0134
Liver cirrhosis	20	(3.09)	233	(3.83)	0.0405	15.7	(2.87)	228.5	(3.77)	0.0519
Connective tissue disease	28	(4.33)	276	(4.54)	0.0103	21.0	(3.82)	273.8	(4.52)	0.0361
Atrial fibrillation	16	(2.47)	245	(4.03)	0.0878	16.5	(3.01)	235.5	(3.89)	0.0496
Peripheral artery disease	33	(5.10)	345	(5.67)	0.0254	20.7	(3.77)	338.8	(5.59)	0.0890
**Hospitalization history (within 3 years before index date)**										
Heart failure	43	(6.65)	653	(10.70)	0.1457	43.6	(7.95)	628.0	(10.40)	0.0867
Myocardial infarction	18	(2.78)	205	(3.37)	0.0341	18.4	(3.36)	202.0	(3.33)	0.0016
Stroke	51	(7.88)	443	(7.29)	0.0225	32.3	(5.89)	443.1	(7.31)	0.0594
Infection	195	(30.10)	2002	(32.90)	0.0600	184.31	(33.60)	1982.13	(32.70)	0.0205
**Medication (within 90 days before index date)**										
ACEi or ARB	452	(69.90)	3712	(61.10)	0.1860	343.5	(62.70)	3746.0	(61.80)	0.0185
Other anti-HTN	598	(92.40)	5701	(93.80)	0.0528	518.6	(94.60)	5678.2	(93.70)	0.0414
Diuretics	590	(91.20)	5514	(90.70)	0.0174	499.9	(91.20)	5502.0	(90.80)	0.0154
Aspirin or Plavix	252	(38.90)	2453	(40.30)	0.0285	209.8	(38.30)	2439.3	(40.30)	0.0421
NSAIDs	166	(25.70)	1229	(20.20)	0.1297	114.3	(20.80)	1252.0	(20.70)	0.0048
Insulin	442	(68.30)	3299	(54.30)	0.2915	331.3	(60.50)	3366.3	(55.50)	0.1036
Sulfonylurea	264	(40.80)	1014	(16.70)	0.5528	109.5	(20.00)	1141.3	(18.80)	0.0305
Acarbose	10	(1.55)	52	(0.86)	0.0634	5.7	(1.04)	55.1	(0.91)	0.0140
Meglitinides	274	(42.30)	1445	(23.80)	0.4028	146.3	(26.70)	1543.9	(25.50)	0.0291
GLP-1	0	(0.00)	1	(0.01)	0.0181	0.0	(0.00)	0.9	(0.02)	0.0176
Anti-cholesterol	281	(43.40)	2721	(44.80)	0.0266	248.4	(45.30)	2707.7	(44.70)	0.0133

ACEi, angiotensin converting enzyme inhibitor; ARB, angiotensin II receptor blocker; ASMD, absolute standardized mean difference; DPP4i, dipeptidyl-peptidase 4 inhibitors; GLP-1, Glucagon-like peptide-1; HTN, hypertension; NSAIDs, non-steroidal anti-inflammatory drugs; PSSW, propensity score method with stabilized weights.

**Table 2:** Incidence rate (per 100 person-year) and hazard ratio (HR) of primary and secondary outcomes for diabetic patients with end-stage renal disease.

	Pioglitazone (*n* = 647)		DPP4i (*n* = 6080)		Cox Results	
	Events	Rate (95% CI)	Events	Rate (95% CI)	HR (95% CI)	*p* Value
Before PSSW						
MACCEs	234	10.18 (8.87–11.48)	1754	12.64 (12.05–13.24)	0.82 (0.71–0.95)	0.0063
All-cause mortality	377	13.07 (11.75–14.39)	2224	13.23 (12.68–13.78)	0.88 (0.78–0.98)	0.0262
Infection related death	226	7.84 (6.81–8.86)	1343	7.99 (7.56–8.42)	0.89 (0.77–1.03)	0.1214
MACCEs related death	117	4.06 (3.32–4.79)	680	4.05 (3.74–4.35)	0.83 (0.67–1.02)	0.0785
After PSSW						
MACCEs	190.3	10.48 (8.99–11.97)	1766.9	12.62 (12.03–13.21)	0.85 (0.73–0.99)	0.0365
All-cause mortality	291.7	12.86 (11.39–14.34)	2247.4	13.22 (12.67–13.77)	0.88 (0.77–0.99)	0.0417
Infection related death	168.3	7.42 (6.30–8.54)	1357.3	7.98 (7.56–8.41)	0.85 (0.72–1.01)	0.0599
MACCEs related death	95.2	4.20 (3.35–5.04)	687.6	4.04 (3.74–4.35)	0.88 (0.70–1.1)	0.2574

CI, confidence interval; DPP4i, dipeptidyl-peptidase 4 inhibitors; HR, hazard ratio; MACCEs, major adverse cardiac cerebrovascular events; PSSW, propensity score method with stabilized weights.

## Supplementary Materials

The following are available online at https://www.mdpi.com/2077-0383/9/11/3578/s1, Figure S1: Kaplan-Meier curve of cumulative incidence for primary and secondary outcomes before propensity score stabilize weighting (PSSW). Table S1. ICD9-code and ICD10-code used in this study. Table S2: Incidence rate (per 100 person-year) and hazard ratio (HR) of MACCEs related outcomes for diabetic patients with end-stage renal disease.
